# CD44 promotes hepatocellular carcinoma progression via upregulation of YAP

**DOI:** 10.1186/s40164-021-00247-w

**Published:** 2021-11-19

**Authors:** Jun Zhang, Xilin He, Yajie Wan, Honghong Zhang, Tao Tang, Meng Zhang, Shiyi Yu, Weiyong Zhao, Liming Chen

**Affiliations:** 1 School of Life Sciences, Nanjing Normal University, Nanjing, China; 2 Department of Oncology, Taizhou People’s Hospital, Taizhou, Jiangsu China; 3 School of Medicine, Yangzhou University, Yangzhou, Jiangsu China; 4 Department of Radiation Oncology, Affiliated Hospital of Integrated Traditional Chinese and Western Medicine, Nanjing University of Chinese Medicine, Nanjing, China; 5 Department of Oncology, Tongren Hospital, Shanghai Jiao Tong University School of Medicine, Shanghai, China

**Keywords:** Hepatocellular carcinoma, CD44, YAP

## Abstract

Hepatocellular carcinoma (HCC) is a common malignancy in human. CD44 is a transmembrane glycoprotein which is frequently overexpressed in cancer of various origins. The function and mechanism of CD44 in HCC remains elusive. In this study, we reported that CD44 was overexpressed in HCC to promote the proliferation and migration of HCC cells via oncogenic YAP, which is the key downstream regulator in Hippo pathway. These findings suggest that CD44-YAP is a probable important axis in pathogenesis of HCC, providing an insight in to HCC pathogenesis as well as potential targets for the intervention of HCC.

**Supplementary Information:**

The online version contains supplementary material available at 10.1186/s40164-021-00247-w.

## Main text

Metastasis and recurrence frequently occur after surgical removal in patients with hepatocellular carcinoma (HCC) [1]. Better understanding on the molecular mechanism behind HCC pathogenesis is required for further development of new therapeutic approaches. CD44 is a multistructural and multifunctional transmembrane glycoprotein [2]. Accumulating evidences reveal dysregulation of CD44 in many types of cancer [3–5]. In this study, we intended to investigate the function and mechanism of CD44 in HCC.

**Fig. 1:**
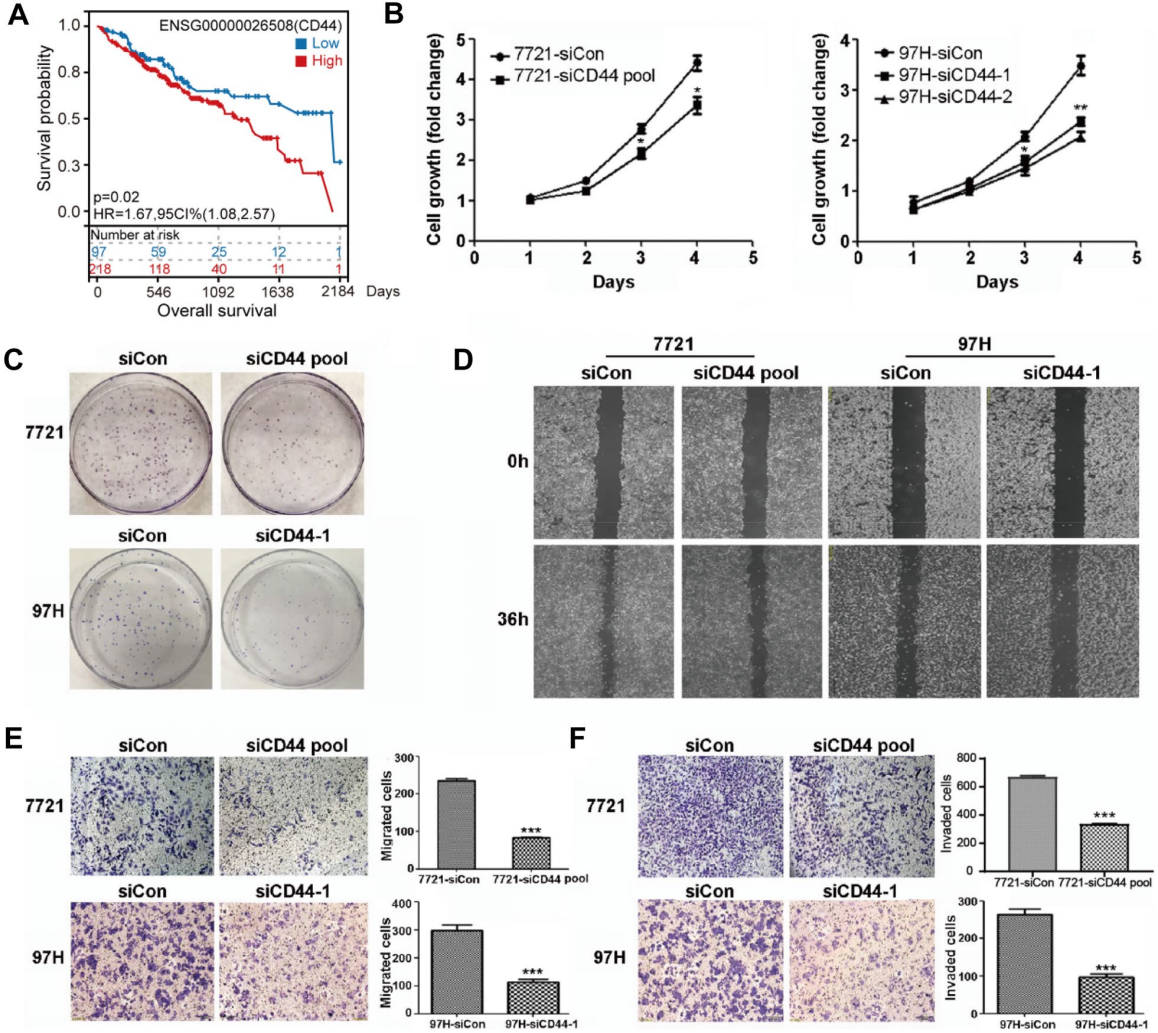
Loss of CD44 inhibited HCC progression. **A** Kaplan–Meier curves show the overall survival of patients with high or low expression of CD44 in HCC. Statistical significance was determined by a log-rank test. **B** Cell viability was measured in SMMC-7721 and MHCC-97 H cells with CD44 knockdown using siRNAs, and the CCK8 assay was performed to analyze cell proliferation. **C**–**F** The colony formation assay, wound-healing, migration and invasion assay were performed in SMMC-7721 and MHCC-97 H cells with CD44 depletion. All data are the mean ± SD, n = 3, *t*-test, *P < 0.05, **P < 0.01, ***P < 0.001

To investigate this, first, we analyzed the expression profile in HCC based on the TCGA database. We found that CD44 expression level was elevated in tumor compared to normal tissues and HCC patients with higher CD44 expression show worse prognosis compared to those with lower CD44 expression (Additional file 1: Fig. S1A, Fig. 1A). In a set of HCC cells lines, SMMC-7721 and MHCC-97 H cells showed high endogenous CD44 expression, while the expression of CD44 in PLC and Huh7 cells was undetectable (Additional file 1: Fig. S1B). To ascertain the role of CD44 in HCC, we silenced CD44 using small interfering RNAs in SMMC-7721 and MHCC-97 H cells, and found that depletion of CD44 significantly inhibited cell proliferation, colony formation, migration and invasion of SMMC-7721 and MHCC-97 H cells (Additional file 1: Fig. 1B–F, Additional file 1: Fig. S1C, D). In agreement with this, ectopic overexpression of CD44 in PLC and Huh7 cells accelerated cell proliferation and enhanced colony forming, migration and invasion ability of cells (Additional file 1: Figs. S1E, S2A–E).

**Fig. 2:**
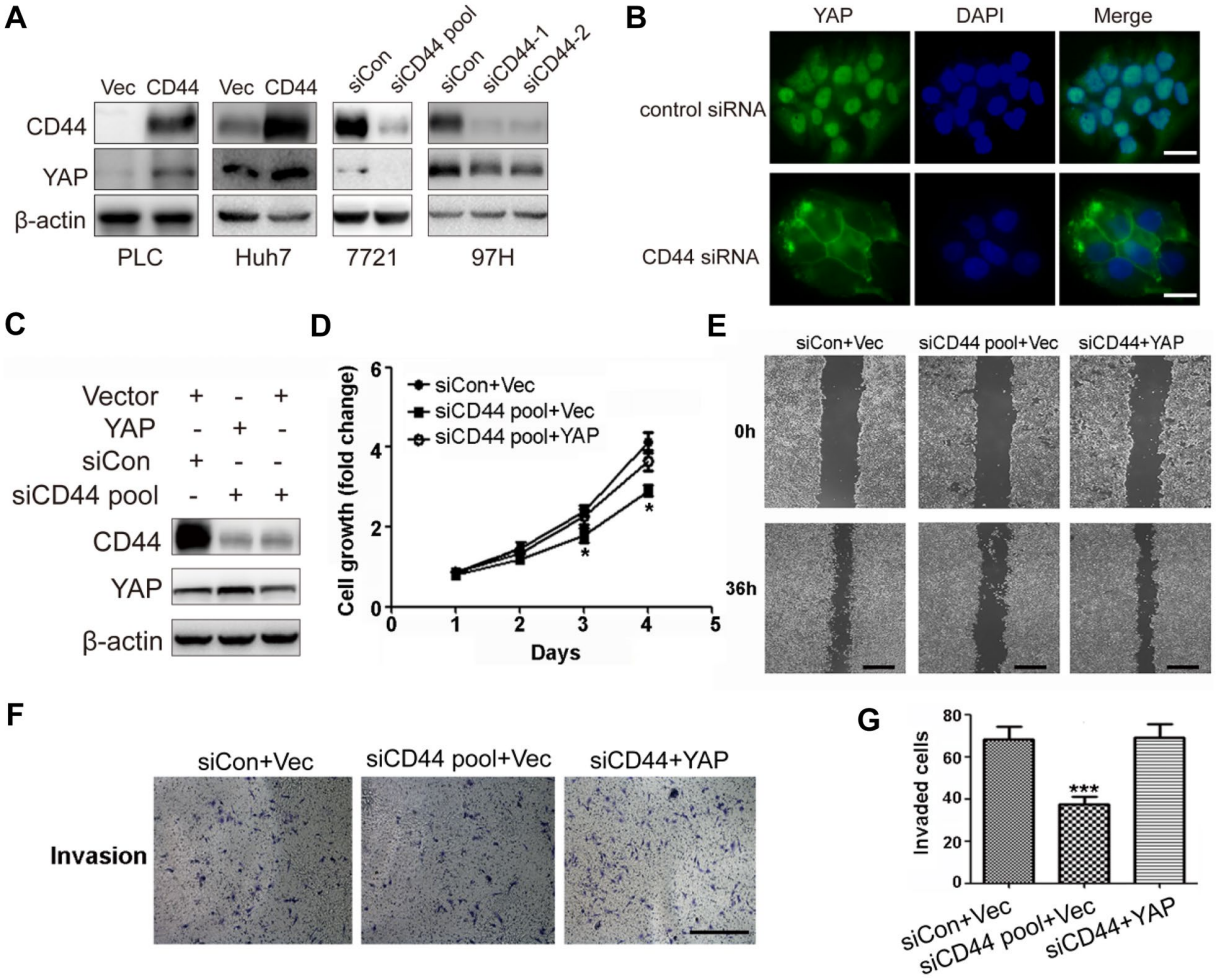
CD44 facilitated HCC progression via regulating Hippo-YAP signaling. **A** Overexpression or knockdown of CD44 in HCC cells, lysates were prepared and blotted with the indicated antibodies. **B** The cellular localization of YAP protein was determined by immunofluorescence in MHCC-97 H cells transfected with CD44 siRNAs, Scale bar represent 20 μm. **C** Representative western blots showed that over-expression of YAP in SMMC-7721 cells with CD44 knockdown. **D–F** Cell viability, wound-healing assay and invasion assay were performed on CD44-depleted SMMC-7721 cells with YAP overexpression, scaled bars represent 400 μm. **G** Quantification of the invaded cells in (**F**). All data are the mean ± SD, n = 3, *t*-test,*P < 0.05, ***P < 0.001

Hippo pathway with oncogenic yes-associated protein (YAP) as the key downstream factor was documented to play an important role in various cancers including HCC via translocating from cytoplasm into nucleus for transcription activation of a set of oncogenic genes [6–8]. We found that YAP protein level was positively regulated by CD44 (Fig. 2A). Additionally, immunofluorescence showed that CD44 silencing led to translocation of YAP from nucleus to cytoplasm (Fig. 2B). Furthermore, when we overexpressed YAP in CD44-depleted HCC cells, we observed that cell proliferation and invasion ability were restored (Fig. 2C–G). These findings suggest that CD44 promoted HCC progression via YAP.

In summary, we demonstrate that overexpression of CD44 promotes HCC progression via YAP. Although loco-regional therapy and systemic chemotherapy are widely used for HCC efficacious treatment, many obstacles still exist for treatment of HCC, in which the most common is drug resistance and recurrence. Therefore, the oncogenic CD44-YAP axis in HCC revealed here can be a potential target for the intervention of HCC.

## Supplementary Information

**Additional file 1.** CD44 promotes hepatocellular carcinoma progression via upregulation of YAP.

## Data Availability

All data generated or analyzed during this study are included in this article are available from the corresponding author on reasonable request.

## Abbreviations

HCC: Hepatocellular carcinoma
YAP: Yes-associated protein
