# Supplementary material for: CD44 promotes hepatocellular carcinoma progression via upregulation of YAP
Source: Exp Hematol Oncol. 2021 Nov 19;10:54. doi: 10.1186/s40164-021-00247-w (PMC8603576; doi:10.1186/s40164-021-00247-w)
Supplement: Supplementary file 1 — Additional file 1. CD44 promotes hepatocellular carcinoma progression via upregulation of YAP. [file 40164_2021_247_MOESM1_ESM.docx]

**Additional file 1**

CD44 promotes hepatocellular carcinoma progression via upregulation of YAP

Jun Zhang^1,#^, Xilin He^1,#^, Yajie Wan^1,#^, Honghong Zhang^2,#^, Tao Tang^1^, Meng Zhang^1^, Shiyi Yu^3,*^, Weiyong Zhao^4, 5,*^, Liming Chen^1,*^

^1^School of Life Sciences, Nanjing Normal University, Nanjing, China.

^2^Department of Oncology, Taizhou People’s Hospital, Taizhou, Jiangsu, China. ^3^School of Medicine, Yangzhou University, Yangzhou, Jiangsu, China.

^4^Department of Radiation Oncology, Affiliated Hospital of Integrated Traditional Chinese and Western Medicine, Nanjing University of Chinese Medicine, Nanjing, China.

^5^Department of Oncology, Tongren Hospital, Shanghai Jiao Tong University School of Medicine, Shanghai, China.

^#^ These authors contributed equally

^*^Correspondence: chenliming1981@njnu.edu.cn; zhaoweiyong976@163.com; [007585@yzu.edu.cn](mailto:007585@yzu.edu.cn)

**Materials and methods**

**Data source and bioinformatics analysis**

The transcriptional profile datasets analyzed in this study were obtained from the Cancer Genome Atlas (TCGA). The total cases are 413, included with Tumor (n=363) and Non-tumor (n=50). Kaplan-Meier method was performed to estimate survival curves based on TCGA database, and the log-rank test was used to compare survival curves of high and low CD44 expression groups.

**Cell culture**

MHCC-97H, SMMC-7721, miha, Huh7 and PLC HCC cell lines were cultured with Dulbecco's Modified Eagle's Media (DMEM) that consists of 10% fetal bovine serum (HyClone; NY, USA) and 1% penicillin-streptomycin solution (PS, Life Technologies) in incubator with 5% CO_2_ at 37°C.

**RNA interference and plasmid transfection**

siControl, siCD44-1 and siCD44-2 were purchased from Genepharma (Suzhou, China). On-target CD44 siRNA and non-targeting control siRNA were purchased from Dharmacon (Lafayette,CO). They were transfected into indicated cell lines using LipofectamineRNAi MAX (Invitrogen; Carlsbad, CA) according to the product brochures, remained for 72 hours and then subjected to protein or RNA extraction.

pCDNA3-YAP-Flag vector which contain full length YAP cDNA was a kind gift from Dr. Zhao Bin. The pcDNA3-YAP-Flag was transfected into HCC cells using Lipofectamine 2000 (Invitrogen) according to the product brochures. Full-length CD44 cDNA was generated by PCR with specific primers and then ligated into pcDNA3-EGFP vector. The pcDNA3-EGFP-CD44 was transfected into indicated HCC cell lines using Lipofectamine 2000 (Invitrogen). The transfected cells were maintained for 48 hours and then subjected to protein lysis or RNA extraction.

For co-transfection of plasmid and siRNA, siRNA was transfected into cells using LipofectamineRNAi MAX. On the next day, plasmid was transfected into cells with Lipofectamine 2000 (Invitrogen). The cells were harvest for western blot or realtime-qPCR 72 hours after siRNA transfection.

**Western blot**

Mouse monoclonal antibody against CD44 (#sc-9960) and rabbit polyclonal antibody against YAP (#sc-101199) were purchased from Santa Cruz Biotechnology. A rabbit antibody against β-actin was purchased from Abclonal (#AC026).Western blotting was carried out following the standard procedure, as described previously.

**Real-time RT-PCR**

RNA was extracted from cell lysates by using the RNeasy kit (Qiagen, Hilden, Germany) and PrimeScript RT reagent kit (TaKaRa, Otsu, Shiga, Japan) was used to synthesize cDNA. The synthesized cDNA was analyzed by quantitative PCR using SYBR Premix Ex Taq (TaKaRa) in a Bio-Rad CFX96 Real-time PCR system (Hercules, CA). GAPDH was used as an endogenous control. The real-time PCR primer sequences are as follows:

*GAPDH* forward, 5’-AGAAGGCTGGGGCTCATTTG-3’

*GAPDH* reverse, 5’-AGGGGCCATCCACAGTCTTC-3’

*YAP* forward, 5’-ACGTTCATCTGGGACAGCAT -3’

*YAP* reverse, 5’-GTTGGGAGATGGCAAAGACA-3’

*CD44* forward, 5’-GGTTACATCTTTTACACCTTTTCTAC-3’

*CD44* reverse, 5’-GAATGTGTCTTGGTCTCTGGTAG-3’.

**Cell proliferation assay and wound-healing assay**

For cell proliferation, HCC cells were seeded into 96-well plates (4×10^5^cells/well) before transfection. After knockdown or overexpression treatment, HCC cells were grown for 24h. 10µl CCK-8(Dojindo Laboratories, Kumamoto, Japan) solution was added into each well and the cells incubated at 37°C for 1h. Then reading the absorbance at 450nm using a Bio-Rad iMark plate reader.

For wound-healing assay, HCC cells were seeded into 6-well plates at a density of 4×10^5^ cells/well. After knockdown or overexpression treatment for 72h, a homogeneous wound was made with a sterile 10µl pipette tip and repeat once in the vertical direction of wound. Subsequent to washing with phosphate buffered saline (PBS), each well was added with 2mL DMEM medium that includes 1% fetal bovine serum (FBS). Then images of each well were captured with microscope (Carl Zeiss Meditec, Jena, Germany) at different time point. All experiments were repeated three times.

#### Transwell migration and invasion assay

Cell migration was assessed by the transwell chamber apparatus (Millipore; Merck KGaA, Darmstadt, Germany). Briefly, the lower chamber was filled with DMEM containing 10% FBS and 5×10^4^ cells in serum-free DMEM were added into the upper chamber with incubation for 24 h at 37°C. Migrated cells on the under surface of Transwells were were fixed with methanol and stained with 1% crystal violet. Images were captured using an inverted microscope and the migrated cells were counted manually. All experiments were repeated 3 times

For cell invasion, cells (1×10^5^ cells/well) were seeded into the Matrigel-coated invasion chambers (Cell Biolabs) with serum-free medium. The lower chamber was filled with DMEM containing 10% FBS and incubation for 24 h. The invading cells were fixed with 4% PFA and stained with 0.5% crystal violet. Images were captured using an inverted microscope and the migrated cells were counted manually. All experiments were repeated 3 times.

**Plate colony formation assay**

Cells were transfected with on-target CD44 siRNA or non-targeting control siRNA and the procedure of transfection is followed above. For plate colony formation assay, cells were seeded into 6-well plate and each well with 500 cells after transfected with CD44 siRNA or non-targeting control siRNA. Then DMEM with 10% fetal bovine serum (HyClone) and 1% PS was added. After culturing for 14 days, colonies were fixed with 4% PFA, stained with Giemsa stain (Leagene) and then the number of the colonies were counted and analyzed.

**Immunofluorescence**

Immunofluorescence assay was performed following the standard procedure, as described previously[[1](#_ENREF_1)]. Rabbit polyclonal anti-YAP was used as primary antibodies and Alexa Fluor 488 secondary antibodies (Invitrogen) was employed to detect fluorescence. The nuclei were stained with DAPI (Vector Laboratories, Cambridgeshire,UK). Representative images were captured using the Leica DM5000 B microscope (Leica Microsystems, Buffalo Grove, IL).

**Statistical analysis**

All the data were presented as the mean ± SEM using three separate experiments. Significant differences were determined using Student t test and one-way variance, performed in GraphPad Prim 7. (*, P<0.05; **, P<0.01; ***, P<0.001)

**References**

1. ZHANG J, HUANG Y, LIU W, LI L, CHEN L. Chaperone-mediated autophagy targeting chimeras (CMATAC) for the degradation of ERα in breast cancer. 2020;44(4):591--95.

**Supplementary Figures**

**Figure S1**

*
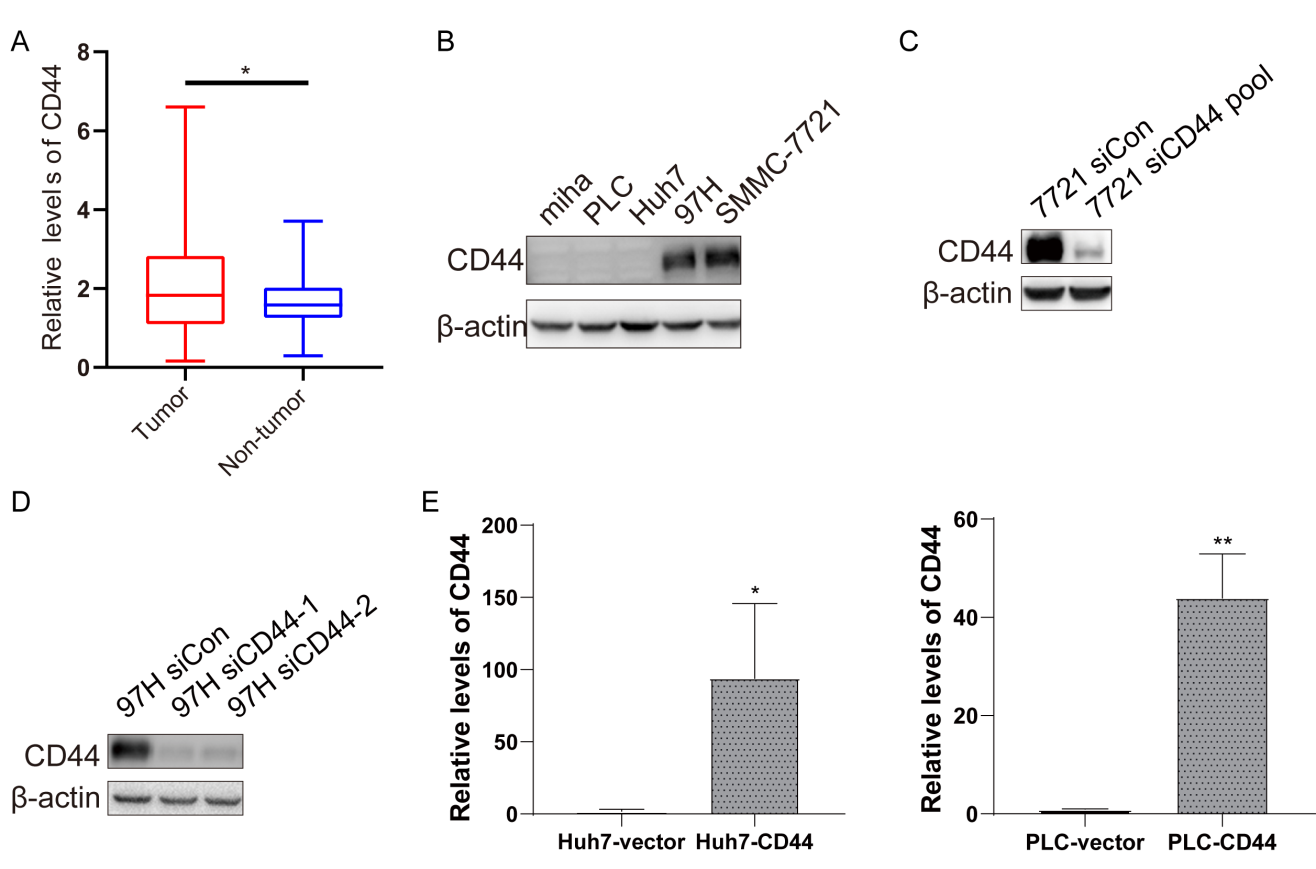
*

Fig. S1 High expression of CD44 was observed in HCC. **A** CD44 expression was elevated in tumor tissues compared with non-tumor tissues base on TCGA database. **B** Western blot analysis for CD44 expression in different HCC cell lines and the immortalized liver cell line miha. **C-D** Representative western blots showed that CD44 was successfully silenced by CD44 siRNAs in SMMC-7721 and MHCC-97H cells. **E** RT-qPCR shows that CD44 was successfully overexpressed in Huh7 and PLC cells upon transfection of recombinant CD44.

**Figure S2**

*
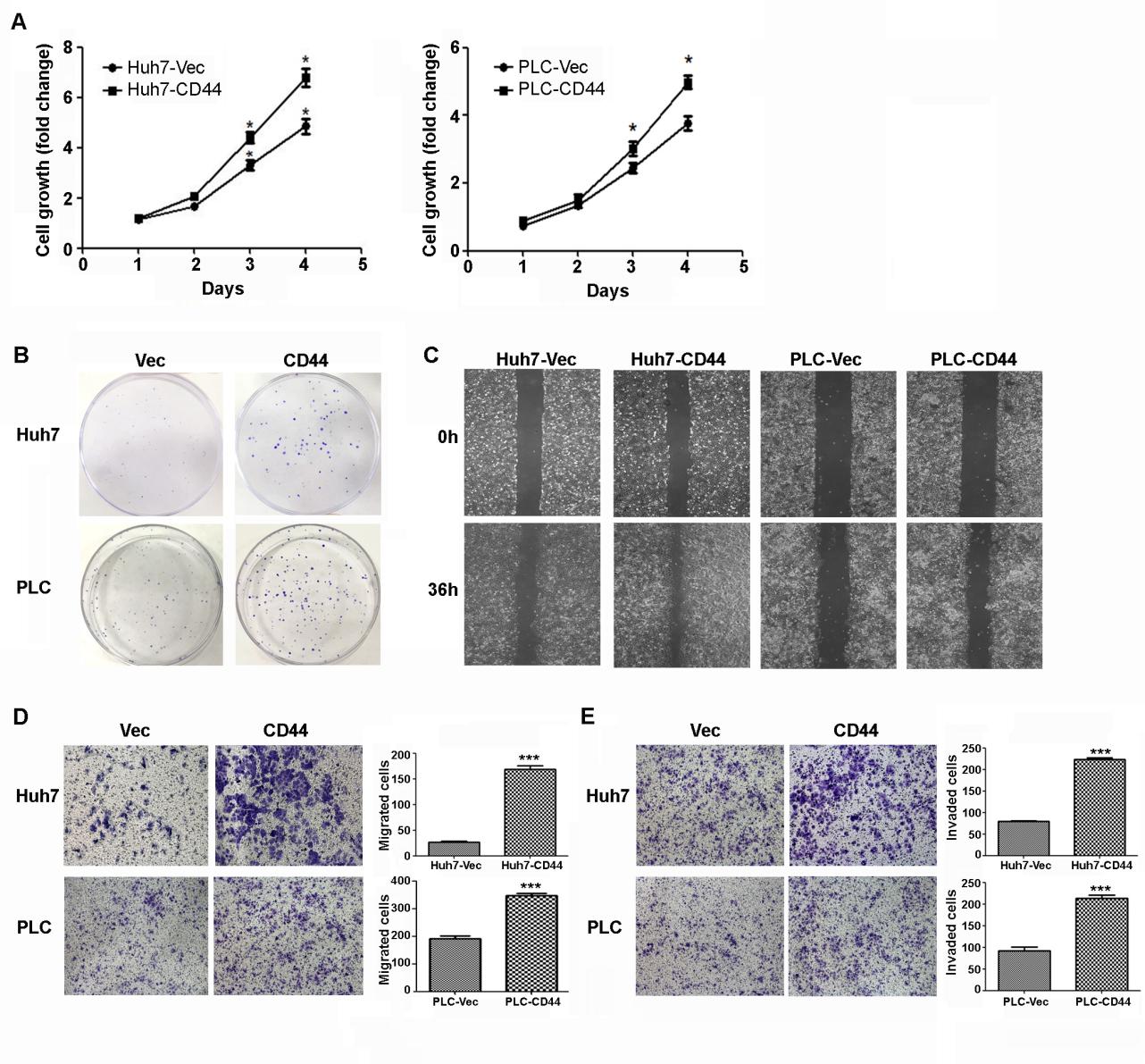
*

Fig. S2 Overexpression of CD44 facilitated HCC progression. **A** Cell proliferation was detected in Huh7 and PLC cells with CD44 overexpression, and the CCK8 assay was used to analyze cell proliferation. **B** The colony formation assays is executed in Huh7 and PLC cells with CD44 overexpression. **C** The wound-healing assay was performed to examine migration abilities of Huh7 and PLC cells with over-expressed CD44, and representative images were taken at 0 and 36 h, scaled bars represent 200μm. **D** The migration assay was performed on Huh7 and PLC cells with CD44 overexpression, scaled bars represent 400μm. **E** The invasion assay was executed on Huh7 and PLC cells with CD44 overexpression, scaled bars represent 400μm. All data are the mean ±SD, n=3, *P < 0.05, ***P < 0.001.
